# The association between joint Serum Neurofilament Light Chain and type 2 diabetes with all-cause and cardiovascular mortality in US adults: a longitudinal study of NHANES

**DOI:** 10.1186/s12902-024-01713-2

**Published:** 2024-09-11

**Authors:** Cuihua Wang, Shuguang Wang, Ying Wang

**Affiliations:** 1 Ultrasound Department, Affiliated Hospital of Shandong Second Medical University, Weifang, Shandong 261000 China; 2 Cardiac Critical Care and Rehabilitation Department, Weifang People’s Hospital, Weifang, Shandong 261000 China; 3 Department of Medical Records Room, Affiliated Hospital of Shandong Second Medical University, Weifang, Shandong 261000 China

**Keywords:** Serum neurofilament light chain, Type 2 diabetes mellitus, Cardiovascular mortality, Longitudinal study, NHANES

## Abstract

**Background:**

In the past, there has been a clear conclusion regarding the sole impact of serum neurofilament light chain (sNfL) levels or type 2 diabetes mellitus (DM) on the risk of death. However, the combined effect of sNfL levels and type 2 DM on all-cause and cardiovascular mortality is still uncertain.

**Methods:**

This study was a prospective cohort study based on data from the National Health and Nutrition Examination Survey (NHANES). The sNfL levels were measured through immunological methods using blood samples collected during the survey. The diagnosis of diabetes was based on rigorous criteria, and participants’ mortality data were followed up until December 31, 2019. Firstly, we separately examined the effects of sNfL and type 2 DM on all-cause and cardiovascular mortality, and finally studied the comprehensive impact of the combination of sNfL and type 2 DM on the risk of mortality. Cumulative Kaplan-Meier curves, multivariate logistic regression and sensitivity analysis were incorporated throughout the entire study.

**Results:**

Participants in the highest quartile of sNfL were observed. Multivariable COX regression model showed that increased sNfL levels and type 2 DM were respectively associated with an increased risk of all-cause and cardiovascular mortality. Furthermore, elevated sNfL levels were significantly associated with an increased risk of all-cause mortality and cardiovascular mortality after adjustment for confounding factors. When considering both elevated sNfL levels and type 2 DM, individuals had a significantly increased risk of mortality. Sensitivity analysis confirmed the robustness of the findings.

**Conclusions:**

These results suggest that elevated levels of sNfL and type 2 DM are associated with an increased risk of all-cause and cardiovascular mortality, and that participants with increased sNfL levels associated with type 2 DM have higher all-cause mortality and cardiovascular mortality.

**Supplementary Information:**

The online version contains supplementary material available at 10.1186/s12902-024-01713-2.

## Introduction

Type 2 diabetes mellitus (DM) is a well-known metabolic disorder characterized by high blood sugar levels and insulin resistance. It has been associated with various complications such as cardiovascular disease, hypertension, and metabolic syndrome [1–3]. In recent years, the incidence of type 2 DM has been rapidly increasing worldwide [4], leading to significant morbidity and mortality and imposing a substantial burden on individuals and healthcare systems.

Neurofilament light chain (NfL) is a protein found in the cellular skeleton of neurons [5]. Previous research has shown that elevated levels of serum NfL (sNfL) are linked to the development of neurodegenerative diseases, as well as sleep disorders, cognitive impairments, and conditions like amyotrophic lateral sclerosis, in which it serves as an indicator of neuronal damage and loss [6–8]. sNfL has also been identified as an important biomarker in diseases related to the nervous system. Studies involving patients with atrial fibrillation have demonstrated significantly higher sNfL levels compared to individuals without this condition [9]. Similarly, in patients with COVID-19, sNfL has emerged as a valuable prognostic indicator [10, 11]. The potential of sNfL as a biomarker lies in its ability to provide real-time information on disease activity, progression, and response to treatment, which is important for early diagnosis, disease monitoring, and assessment of treatment. Recent studies have indicated that sNfL exhibits higher levels in patients with type 2 DM and may be associated with cardiovascular mortality. In cardiovascular disease, increased levels of sNfL may reflect the presence of microvascular lesions and neuroinflammation, both of which are associated with an increased risk of adverse cardiovascular events [12]. However, there is currently limited research exploring the association between sNfL levels and the risk of developing type 2 DM, as well as the impact of combined changes in sNfL and type 2 DM on mortality.

Although the impact of sNfL and type 2 DM on mortality has been elucidated, their combined effect on all-cause and cardiovascular mortality is still unclear. In our study, we hypothesised that sNfL plays a synergistic role with type 2 DM on mortality. Therefore, the primary aim of this study is to examine the potential impact of changes in sNfL levels combined with the presence of type 2 DM on the risk of mortality. By exploring these associations, we hope to contribute to a better understanding of the role of sNfL in type 2 DM and its potential implications for mortality.

## Methods

### Data sources

This study is based on the data obtained from National Health and Nutrition Examination Survey (NHANES), which is a nationally representative survey conducted in the United States. NHANES utilizes a stratified, multistage, probability sampling design to select participants from various demographic groups. The survey collects demographic information, health status, dietary surveys, physical examinations, and laboratory test results. For this study, we utilized data from standardized questionnaires administered to the participants, as well as corresponding health indicator data relevant to our research objectives. In order to assess mortality, participant data was linked with the National Death Index, which provides information on deaths in the United States. This study was approved by the NCHS Ethics Review Board (ERB) under Ethics Approval Number: continuation of protocol #2011-17. All participants provided written informed consent prior to participation in the study.

### Study population

This study utilized survey data from the years 2013 to 2014. The initial sample included a total of 10,175 participants. The participants’ mortality data were followed up until December 31, 2019. To ensure the study’s relevance to the adult population, individuals under the age of 20 were excluded from the analysis. After applying the age exclusion criterion and accounting for missing information related to type 2 DM and sNfL levels, 2,053 participants were included in the final analysis. The specific criteria used for inclusion and exclusion can be found in Figure S1, which provides a visual representation of the participant selection process.

### Primary variables

In this study, the sNfL levels were measured using a highly sensitive immunological method. The remaining or original serum samples from the NHANES 2013–2014 were utilized and tested using an automated platform to determine the levels of sNfL. Further information and details about the measurement method can be found in the provided link: https://wwwn.cdc.gov/Nchs/Nhanes/2013-2014/SSSNFL_H.htm.

In our study, the diagnosis of type 2 DM was strictly defined. The diagnosis was determined based on several criteria, including self-report of type 2 DM, use of insulin or oral hypoglycemic medication, fasting blood glucose (FBG) levels of 126 mg/dL or higher, or HbA1c (glycated hemoglobin) levels of 6.5% or higher [13].

### Assessment of mortality

Mortality data for this analysis were obtained from the NHANES Public Use Link Mortality File and integrated with the standard NHANES dataset using unique respondent serial numbers assigned to each participant. The cardiovascular deaths were identified using the 10th revision of the International Statistical Classification of Diseases codes I00-I09, I11, I13, and I20-I51, which encompass a range of cardiovascular conditions.In our analysis of all-cause mortality, we included all instances of death among participants during the follow-up period, without regard to the specific underlying cause of death. This approach provides a comprehensive assessment of the impact of elevated sNfL levels and type 2 diabetes on the overall risk of mortality.

### Covariate

We rigorously accounted for confounding variables in our study by considering the following factors: age, sex, race, education, family poverty index (PIR), drinking status, smoking status, body mass index (BMI), Physical activity, hypertension, cardiovascular disease, and neurologic diseases. Specifically, age was stratified into 60-year intervals. Race was subdivided into non-Hispanic Whites, non-Hispanic Blacks, and other races. Education level was categorized as below high school, high school, and above high school. Moreover, family PIR was divided into three groups based on two thresholds at 1.0 and 3.0. To account for drinking status, participants were classified as nondrinkers, low to moderate drinkers (defined as males consuming less than 2 drinks per day and females consuming less than 1 drink per day), or heavy drinkers (defined as males consuming 2 or more drinks per day and females consuming 1 or more drink per day). Furthermore, smoking status was categorized as current, former, or never smoker. BMI was classified as under 25 kg/m^2^, overweight (25–29.9 kg/m^2^), or obese (over 29.9 kg/m^2^). Physical activity was classified as inactive, insufficiently active or active. In addition, we collected data on cardiovascular disease, hypertension, and neurologic diseases through NHANES questionnaire items.

### Statistical analysis

To account for the complex sampling design of NHANES and ensure the representativeness of the estimates, survey weights were applied to all calculations and analysis. Continuous variables without a normal distribution are presented as medians (interquartile ranges). Categorical variables are presented as numbers (percentages). Cumulative Kaplan-Meier (KM) curves were plotted for all-cause and cardiovascular mortality based on sNfL levels and the presence of type 2 DM. Log-rank tests were conducted to compare the differences between various groups. The Cox proportional hazards model is used to evaluate the association between sNfL and type 2 DM, as well as their combined effect, with mortality. The hazard ratio (HR) and its 95% confidence interval (95%CI) were used to record the results. We utilized two different models: crude model and model 1 adjusted for age, sex, and race. To assess the robustness of the results, sensitivity analysis were performed, excluding individuals with cancer history, individuals with neurologic diseases, and individuals with cardiovascular disease.

The statistical analysis of the data was performed using the R Language and Environment for Statistical Computing (version 4.1.3). A *p*-value threshold of less than 0.05 was considered statistically significant.

## Results

### Baseline characteristics

The study included a total of 2053 participants, and people over 60 years old accounted for 21.30%. The baseline characteristics of the participants are presented in Table 1, stratified by quartiles of sNfL levels. Participants in the highest quartile of sNfL were observed to be older, with higher proportions of males, non-Hispanic whites, smokers, obese, and those with hypertension, cardiovascular disease, and neurological disease (*P*<0.05). In addition, they had higher all-cause and cardiovascular mortality (*P*<0.05).

**Table 1:** Baseline characteristics of the general adult population according to quartiles of sNfL in NHANES 2013–2014

Characteristics	Total	Quartiles of sNfL				*P* value
		< 8.2	8.3–12.3	12.4–19.1	> 19.1	
Participants	2053	516	516	509	512	
Age, years						< 0.001
20–59	1511 (78.70)	509 (99.56)	435 (86.97)	327 (70.77)	240 (55.26)	
≥60	542 (21.30)	7 (0.44)	81 (13.03)	182 (29.23)	272 (44.74)	
Sex, %						0.005
Female	1063 (50.85)	301 (56.65)	262 (49.31)	258 (51.38)	242 (45.59)	
Male	990 (49.15)	215 (43.35)	254 (50.69)	251 (48.62)	270 (54.41)	
Race/ethnicity, %						< 0.001
Non-Hispanic White	904 (65.11)	192 (53.83)	211 (63.01)	248 (72.08)	253 (72.50)	
Non-Hispanic Black	367 (11.84)	91 (13.70)	107 (13.88)	70 (8.13)	99 (11.45)	
Other race	782 (23.05)	233 (32.47)	198 (23.11)	191 (19.79)	160 (16.04)	
Education level, %						0.619
Below high school	451 (15.83)	113 (17.95)	108 (15.10)	100 (14.21)	130 (15.94)	
High school	429 (20.19)	98 (18.56)	118 (22.30)	100 (19.35)	113 (20.58)	
Above high school	1173 (63.98)	305 (63.49)	290 (62.60)	309 (66.44)	269 (63.47)	
Family PIR, %						0.123
≤ 1.0	506 (18.38)	135 (20.92)	126 (18.66)	119 (17.73)	126 (15.96)	
1.1–3.0	767 (33.17)	195 (37.41)	183 (30.60)	184 (29.45)	205 (35.08)	
> 3.0	780 (48.45)	186 (41.67)	207 (50.74)	206 (52.82)	181 (48.96)	
Drinking status, %						0.222
Nondrinker	374 (15.47)	87 (15.32)	84 (14.21)	102 (15.75)	101 (16.66)	
Low-to-moderate drinker	1509 (74.97)	394 (77.11)	394 (78.36)	357 (72.37)	364 (71.70)	
Heavy drinker	170 (9.57)	35 (7.57)	38 (7.43)	50 (11.88)	47 (11.64)	
Smoking status						0.024
Never smoker	1141 (56.15)	322 (63.73)	305 (58.31)	265 (52.24)	249 (49.57)	
Former smoker	458 (22.45)	82 (15.64)	108 (22.03)	133 (25.37)	135 (27.34)	
Current smoker	454 (21.41)	112 (20.63)	103 (19.66)	111 (22.39)	128 (23.10)	
BMI, %						0.007
< 25.0 kg/m^2^	621 (29.33)	148 (27.48)	177 (32.87)	167 (32.80)	129 (24.03)	
25.0–29.9 kg/m^2^	652 (32.55)	141 (27.87)	159 (33.17)	185 (36.90)	167 (32.54)	
> 29.9 kg/m^2^	780 (38.13)	227 (44.65)	180 (33.96)	157 (30.30)	216 (43.43)	
Physical activity						0.044
Inactive	487 (22.77)	110 (20.19)	92 (17.66)	126 (23.29)	159 (30.48)	
Insufficiently active	673 (34.68)	190 (40.19)	163 (32.71)	148 (29.40)	172 (36.16)	
Active	893 (42.55)	216 (39.62)	261 (49.64)	235 (47.31)	181 (33.36)	
Hypertension, %						< 0.001
No	1223 (62.77)	404 (81.35)	331 (65.55)	279 (58.33)	209 (44.08)	
Yes	830 (37.23)	112 (18.65)	185 (34.45)	230 (41.67)	303 (55.92)	
Cardiovascular diseases, %						< 0.001
No	1888 (92.93)	510 (99.24)	492 (94.59)	461 (92.15)	425 (85.06)	
Yes	165 (7.07)	6 (0.76)	24 (5.41)	48 (7.85)	87 (14.94)	
Neurologic diseases, %	130 (5.03)	10 (1.68)	13 (2.62)	37 (5.78)	70 (10.49)	< 0.001
Parkinson’s disease	16 (0.74)	5 (0.80)	1 (0.09)	1 (0.34)	9 (1.79)	
Epilepsy	15 (0.79)	4 (0.80)	4 (0.74)	3 (0.70)	4 (0.92)	
Cognitive impairment	55 (1.39)	1 (0.05)	3 (0.21)	15 (1.51)	36 (3.97)	
Stroke	52 (2.43)	1 (0.18)	6 (1.66)	19 (3.74)	26 (4.38)	
type 2 DM, %						< 0.001
No	1688 (85.72)	478 (94.10)	456 (90.46)	410 (83.91)	344 (73.42)	
Yes	365 (14.28)	38 (5.90)	60 (9.54)	99 (16.09)	168 (26.58)	
NfL, pg/mL	12.1 [8.1, 18.6]	6.2 [5.1, 7.2]	10.2 [9.2, 11.2]	14.9 [13.7, 16.7]	27.4 [22.5, 37.3]	< 0.001
All-cause mortality, %						< 0.001
No	1970 (96.56)	515 (99.82)	503 (98.00)	485 (96.06)	467 (91.99)	
Yes	83 (3.44)	1 (0.18)	13 (2.00)	24 (3.94)	45 (8.01)	
Cardiovascular mortality, %						< 0.001
No	2036 (99.34)	516 (100.00)	514 (99.79)	505 (99.62)	501 (97.85)	
Yes	17 (0.66)	0 (0.00)	2 (0.21)	4 (0.38)	11 (2.15)	

Abbreviations: sNfL, serum neurofilament light chain; BMI, Body mass index; DM, diabetes mellitus; PIR, poverty income ratio. Continuous variables without a normal distribution are presented as medians [interquartile ranges]. Categorical variables are presented as numbers (percentages). Sampling weights were applied for calculation of demographic descriptive statistics; N reflect the study sample while percentages reflect the survey-weighted data

Participants were also divided into two groups based on the presence or absence of type 2 DM (Table S1). Participants with type 2 DM were older, had a higher proportion of non-drinkers, higher smoking cessation rates, higher obesity rates, lower physical activity rates, and a higher prevalence of hypertension, cardiovascular diseases, and neurological disorders (*P*<0.05). Participants with type 2 DM also had higher all-cause and cardiovascular mortality (*P*<0.05).

### Association between single sNfL or type 2 DM and mortality

**Fig. 1:**
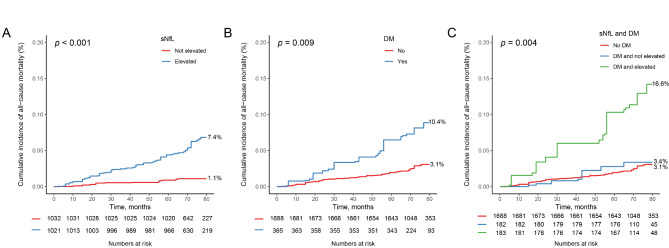
Kaplan-Meier cumulative survival curves for the overall mortality based on the levels of sNfL (**A**), type 2 DM (**B**), and the combination of both (**C**)

**Fig. 2:**
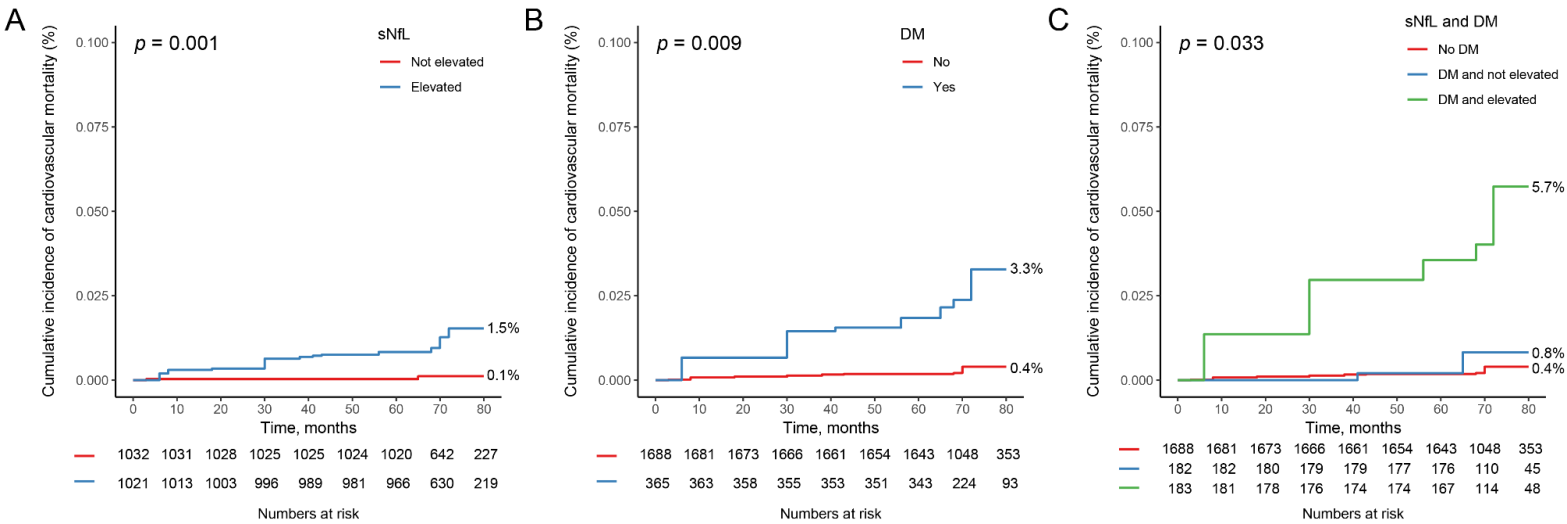
Kaplan-Meier cumulative survival curves for the cardiovascular mortality based on the levels of sNfL (**A**), type 2 DM (**B**), and the combination of both (**C**)

Figures 1A and 2B depicts the KM survival curves based on the levels of sNfL. It is evident that subjects with elevated sNfL faced significantly increased cumulative risks of all-cause and cardiovascular mortality compared to those with non-elevated sNfL (*P* < 0.001, *P* = 0.001, respectively). The association between sNfL levels and mortality risk was assessed using a multivariable Cox regression analysis (Table 2). After adjusting for confounding factors, the sNfL levels showed a positive association with all-cause and cardiovascular mortality. (HR = 3.04, 95%CI: 2.14–4.33, HR = 4.70, 95%CI: 3.26–6.77, respectively). Further divide participants into non-elevated and elevated group base on sNfL levels, the elevated sNfL significantly increased all-cause and cardiovascular mortality (HR = 4.38, 95%CI: 2.04–9.39, HR = 10.61, 95%CI: 2.05-55.00, respectively).

**Table 2:** Association between sNfL levels and type 2 DM and their combined effect on all-cause mortality

	Crude			Model 1	
	HR (95% CI)	*P* value		HR (95% CI)	*P* value
sNfL levels					
Continuous sNfL	3.21(2.47,4.18)	< 0.001		3.04(2.14,4.33)	< 0.001
Not elevated	1 [Reference]			1 [Reference]	
Elevated	5.60(2.50,12.53)	< 0.001		4.38(2.04,9.39)	< 0.001
type 2 DM					
No	1 [Reference]			1 [Reference]	
Yes	3.13(1.48,6.60)	0.003		2.49(1.23,5.05)	0.011
sNfL and type 2 DM					
No type 2 DM	1 [Reference]			1 [Reference]	
type 2 DM and not elevated	1.25(0.50, 3.11)	0.626		1.14(0.45,2.93)	0.778
type 2 DM and elevated	5.10(2.39,10.89)	< 0.001		3.79(1.88,7.65)	< 0.001

Abbreviations: HR, hazard ratio; CI, confidence interval; sNfL, serum neurofilament light chain; DM, diabetes mellitus; Model 1 was adjusted for age (20–59 or ≥ 60), sex (male or female), and race (Non-Hispanic White, Non-Hispanic Black or Other). Elevated sNfL is characterized by values exceeding the median sNfL. Elevated sNfL is characterized by values exceeding the median sNfL

Moreover, Figs. 1B and 2B indicates that participants with type 2 DM exhibited significantly higher cumulative risks of all-cause and cardiovascular mortality in comparison to non-diabetic participants (*P* = 0.009, *P* = 0.009, respectively). And type 2 DM also increased both all-cause and cardiovascular mortality among participants (HR = 2.49, 95%CI: 1.23–5.05, HR = 8.01, 95%CI: 1.99–32.24, respectively).

### Combined association between sNfL and type 2 DM and mortality

Furthermore, the impact of sNfL combined with type 2 DM on mortality was assessed in Figs. 1C and 2C through the KM curves, which demonstrated a notably heightened risk of death in individuals with elevated sNfL levels and type 2 DM (all-cause: *P* = 0.004, cardiovascular: *P* = 0.033). To explore the independent effect of sNfL combined with type 2 DM on mortality, Tables 2 and 3 were utilized. Compared to non-diabetic individuals with no elevated sNfL levels, individuals with elevated sNfL levels and Type 2 DM had a significantly increased risk of all-cause mortality and cardiovascular mortality (HR = 3.79, 95%CI: 1.88–7.65, HR = 14.35, 95%CI: 2.79–73.92, respectively).

**Table 3:** Association between sNfL levels and type 2 DM and their combined effect on cardiovascular mortality

	Crude			Model 1	
	HR (95% CI)	*P* value		HR (95% CI)	*P* value
sNfL levels					
Continuous sNfL	4.36 (2.91–6.55)	< 0.001		4.70 (3.26–6.77)	< 0.001
Not elevated	1 [Reference]			1 [Reference]	
Elevated	12.36 (2.66,57.54)	0.001		10.61 (2.05,55.00)	0.005
type 2 DM					
No	1 [Reference]			1 [Reference]	
Yes	8.78 (2.80-27.54)	< 0.001		8.01 (1.99–32.24)	0.003
sNfL and type 2 DM					
No type 2 DM	1 [Reference]			1 [Reference]	
type 2 DM and not elevated	2.31 (0.39,13.53)	0.353		2.45 (0.42,14.30)	0.319
type 2 DM and elevated	15.54 (4.80-50.35)	< 0.001		14.35 (2.79–73.92)	0.001

Abbreviations: HR, hazard ratio; CI, confidence interval; sNfL, serum neurofilament light chain; DM, diabetes mellitus; Model 1 was adjusted for age (20–59 or ≥ 60), sex (male or female), and race (Non-Hispanic White, Non-Hispanic Black or Other). Elevated sNfL is characterized by values exceeding the median sNfL

### Sensitivity analysis

Sensitivity analysis were conducted to confirm the robustness of the findings. After excluding participants who had cancer history, the combined effect of sNfL and type 2 DM increased the all-cause and cardiovascular mortality risk (HR = 5.00, 95%CI: 2.25–11.10, HR = 70.42, 95%CI: 29.53-167.93, respectively). Similarly, after excluding participants with neurologic diseases and those with a history of cardiovascular disease, the combined effect of sNfL and type 2 DM remained a risk factor for mortality (Table S2–4).

## Discussion

This study, conducted through large-scale population analysis, suggests that an increase in sNfL levels and type 2 DM are respectively associated with increased all-cause and cardiovascular mortality. Furthermore, the elevated levels of sNfL combined with type 2 DM are independently associated with higher all-cause and cardiovascular mortality. Sensitivity analysis further confirms the stability of these findings.

sNfL is a protein present in neurons that is released into the bloodstream when the nervous system is damaged [14]. Previous research has found an association between Type 2 DM and elevated sNfL levels, this indicates that the level of sNfL is more significant in the population of type 2 diabetes patients [15]. Therefore, solely focusing on the research of type 2 DM’s impact on all-cause and cardiovascular mortality is insufficient and unreasonable.

Furthermore, increased sNfL levels are also associated with higher mortality. A study in the general population of the United States found a clear association between increasing sNfL levels and higher mortality [16]. Another study conducted in Parkinson’s disease patients found a correlation between elevated sNfL levels and increased mortality [17]. Research on patients with COVID-19 has also revealed that COVID-19 increases sNfL levels, which further exacerbates in-hospital mortality [18]. In addition, sNfL has been found to be a biomarker of long-term outcome in diseases such as polyneuropathy, multiple sclerosis, ischaemic stroke and traumatic brain injury [19–22]. In our study, we observed a significant increase in the all-cause and cardiovascular mortality among elevated sNfL participants, which is consistent with previous research. In addition, we also emphasized the combined effect of elevated sNfL and type 2 DM on all-cause and cardiovascular mortality and found that the former can significantly increase the risk of mortality. There is no doubt that this study fills in some gaps of previous research.

In sensitivity analysis, we excluded individuals with major diseases such as cancer, cardiovascular diseases, and neurological disorders, further verifying the robustness of our findings. This enhances the feasibility of using sNfL as a potential predictive tool in the future.

The association between sNfL levels and type 2 DM and mortality may be explained through inflammatory mechanisms. Studies have found that elevated sNfL levels are associated with neuroinflammation [23, 24]. In individuals with type 2 DM and elevated sNfL levels, higher levels of inflammation further contribute to increased all-cause and cardiovascular mortality. Our findings suggest that elevated sNfL levels and type 2 DM not only independently increase all-cause and cardiovascular mortality, but that their combined effect further enhances this risk.

The relationship between sNfL levels, type 2 DM cardiovascular risk, and mortality fundamentally involves interactions between the nervous, metabolic, and cardiovascular systems. Given the potential role of sNfL in diagnosing and predicting various neurological disorders, elevated sNfL levels may be associated with dysfunctions in the insular cortex and other autonomic nerve regulating cardiac activity. Additionally, persistent and abnormal inflammation is considered a major contributor to adverse outcomes [25]. Neuroinflammation, characterized primarily by the activation of microglia, involves M1 microglia producing pro-inflammatory cytokines (TNF-α, IL-6, andIL-1β), chemokines, and reactive oxygen species (ROS), leading to acute immune responses [26]. IL-1, in particular, can trigger adverse remodeling, heart failure, and arrhythmias [27]. Mediators such as the P2 × 7 purinergic receptor (P2 × 7R) and macrophage-induced type C lectin (Mincle) are thought to play significant regulatory roles in this context [26, 28]. Furthermore, metabolic disturbances caused by type 2 DM exacerbate neurological damage and inflammation, contributing to adverse outcomes.

Although this study utilized large-scale survey data from the United States and accounted for potential confounding factors, there are still limitations to consider. Firstly, we only measured sNfL levels once, which may differ from the true level. Therefore, further measurements are needed to determine the stable level of sNfL. Secondly, while we considered potential confounding factors, there may still be unaccounted factors, so caution should be exercised in interpreting the results. Finally, this study was conducted on a population in the United States, and it remains to be determined whether the findings can be generalized to other populations through large-scale studies.

## Conclusions

In conclusion, the elevation of sNfL levels and type 2 DM not only individually increase the risks of all-cause and cardiovascular mortality, but their combined effect further enhances the mortality. This finding highlights the potential value of sNfL as a potential biomarker in the management of diabetes and its complications. In addition, our findings may contribute to the development of new preventive and therapeutic strategies to reduce mortality in patients with type 2 DM.

## Electronic supplementary material

Below is the link to the electronic supplementary material.

Supplementary Material 1

## Data Availability

Publicly available datasets were analyzed in this study. This data can be found here: https://www.cdc.gov/nchs/nhanes/.

## Abbreviations

NHANES: National Health and Nutritional Examination Survey
CI: Confidence interval
sNfL: Serum neurofilament light chain
DM: Diabetes mellitus
PIR: Family poverty index
BMI: Body mass index
HR: Hazard ratio
