# Supplementary material for: The association between joint Serum Neurofilament Light Chain and type 2 diabetes with all-cause and cardiovascular mortality in US adults: a longitudinal study of NHANES
Source: BMC Endocr Disord. 2024 Sep 11;24:186. doi: 10.1186/s12902-024-01713-2 (PMC11389518; doi:10.1186/s12902-024-01713-2)
Supplement: Supplementary file 1 — Supplementary Material 1 [file 12902_2024_1713_MOESM1_ESM.docx]

**Online Supplementary Material**

**Figure S1.** Flow chart of the study.

**Table S1.** Baseline characteristics of the general adult population according to type 2 DM in NHANES 2013–2014.

**Table S2.** Association between sNfL levels and type 2 DM and their combined effect on mortality after excluding participants who had cancer history at baseline in NHANES 2013–2014 (n=1896).

**Table S3.** Association between sNfL levels and type 2 DM and their combined effect on mortality after excluding participants who had neurologic diseases history at baseline in NHANES 2013–2014 (n=1923).

**Table S4.** Association between sNfL levels and type 2 DM and their combined effect on mortality after excluding participants who had CVD history at baseline in NHANES 2013–2014 (n=1888).

**
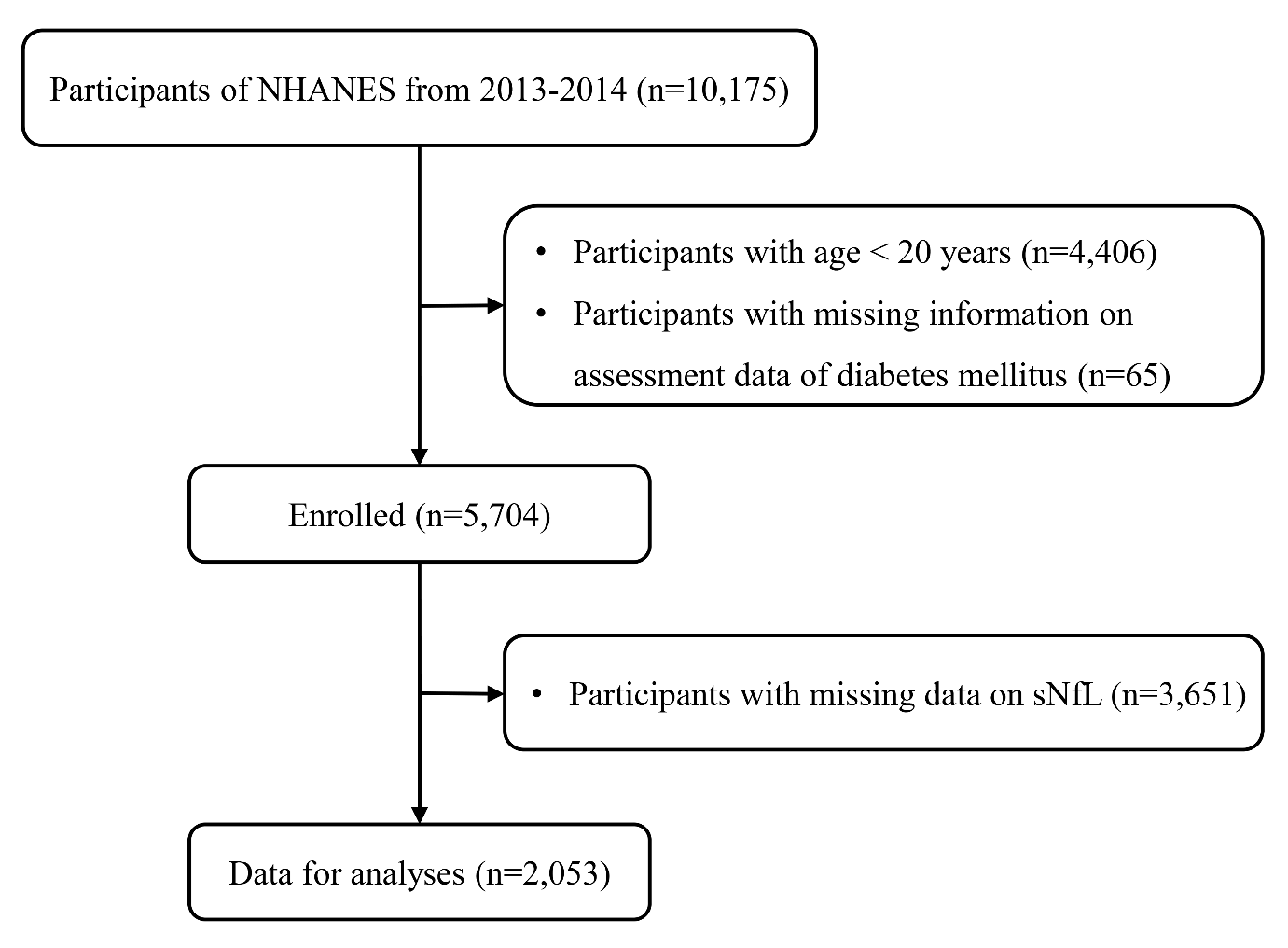
**

**Figure S1.** Flow chart of the study.

**Table S1.** Baseline characteristics of the general adult population according to type 2 DM in NHANES 2013–2014.

| **Characteristics** | **Total (n=2053)** | **type 2 DM** | | ***P* value** |
| --- | --- | --- | --- | --- |
|  |  | **No (n=1688)** | **Yes (n=365)** |  |
| Age, years |  |  |  | <0.001 |
| 20-59 | 1511 (78.70) | 1334 (82.33) | 177 (56.86) |  |
| ≥60 | 542 (21.30) | 354 (17.67) | 188 (43.14) |  |
| Sex, % |  |  |  | 0.754 |
| Female | 1063 (50.85) | 879 (51.04) | 184 (49.71) |  |
| Male | 990 (49.15) | 809 (48.96) | 181 (50.29) |  |
| Race/ethnicity, % |  |  |  | 0.537 |
| Non-Hispanic White | 904 (65.11) | 762 (65.38) | 142 (63.45) |  |
| Non-Hispanic Black | 367 (11.84) | 291 (11.54) | 76 (13.66) |  |
| Other race | 782 (23.05) | 635 (23.08) | 147 (22.89) |  |
| Education level, % |  |  |  | 0.262 |
| Below high school | 451 (15.83) | 348 (15.19) | 103 (19.68) |  |
| High school | 429 (20.19) | 345 (19.75) | 84 (22.81) |  |
| Above high school | 1173 (63.98) | 995 (65.06) | 178 (57.51) |  |
| Family PIR, % |  |  |  | 0.894 |
| ≤1.0 | 506 (18.38) | 412 (18.46) | 94 (17.90) |  |
| 1.1–3.0 | 767 (33.17) | 616 (32.94) | 151 (34.57) |  |
| >3.0 | 780 (48.45) | 660 (48.60) | 120 (47.53) |  |
| Drinking status, % |  |  |  | 0.013 |
| Nondrinker | 374 (15.47) | 283 (14.57) | 91 (20.86) |  |
| Low-to-moderate drinker | 1509 (74.97) | 1254 (75.11) | 255 (74.13) |  |
| Heavy drinker | 170 (9.57) | 151 (10.33) | 19 (5.01) |  |
| Smoking status |  |  |  | 0.001 |
| Never smoker | 1141 (56.15) | 957 (57.16) | 184 (50.05) |  |
| Former smoker | 458 (22.45) | 344 (20.83) | 114 (32.18) |  |
| Current smoker | 454 (21.41) | 387 (22.01) | 67 (17.77) |  |
| BMI, % |  |  |  | <0.001 |
| <25.0 kg/m^2^ | 621 (29.33) | 580 (32.80) | 41 (8.48) |  |
| 25.0-29.9 kg/m^2^ | 652 (32.55) | 530 (32.37) | 122 (33.59) |  |
| >29.9 kg/m^2^ | 780 (38.13) | 578 (34.83) | 202 (57.93) |  |
| Physical activity |  |  |  | <0.001 |
| Inactive | 487 (22.77) | 368 (20.44) | 119 (36.79) |  |
| Insufficiently active | 673 (34.68) | 554 (34.85) | 119 (33.69) |  |
| Active | 893 (42.55) | 766 (44.72) | 127 (29.52) |  |
| Hypertension, % |  |  |  | <0.001 |
| No | 1223 (62.77) | 1106 (67.85) | 117 (32.28) |  |
| Yes | 830 (37.23) | 582 (32.15) | 248 (67.72) |  |
| Cardiovascular diseases, % |  |  |  | <0.001 |
| No | 1888 (92.93) | 1599 (95.35) | 289 (78.39) |  |
| Yes | 165 (7.07) | 89 (4.65) | 76 (21.61) |  |
| Neurologic diseases, % | 130 (5.03) | 80 (4.04) | 50 (10.99) | <0.001 |
| Parkinson’s disease | 16 (0.74) | 10 (0.62) | 6 (1.51) |  |
| Epilepsy | 15 (0.79) | 14 (0.77) | 1 (0.90) |  |
| Cognitive impairment | 55 (1.39) | 28 (0.99) | 27 (3.76) |  |
| Stroke | 52 (2.43) | 34 (1.88) | 18 (5.78) |  |
| sNFL, pg/mL | 12.10 [8.10,18.60] | 11.40 [7.70,17.30] | 17.60 [11.90,27.70] | <0.001 |
| All-cause mortality, % |  |  |  | 0.007 |
| No | 1970 (96.56) | 1634 (97.35) | 336 (91.82) |  |
| Yes | 83 (3.44) | 54 (2.65) | 29 (8.18) |  |
| Cardiovascular mortality, % |  |  |  | <0.001 |
| No | 2036 (99.34) | 1679 (99.68) | 357 (97.27) |  |
| Yes | 17 (0.66) | 9 (0.32) | 8 (2.73) |  |

Abbreviations: sNfL, serum neurofilament light chain; BMI, Body mass index; DM, diabetes mellitus; PIR, poverty income ratio. Continuous variables without a normal distribution are presented as medians [interquartile ranges]. Categorical variables are presented as numbers (percentages). Sampling weights were applied for calculation of demographic descriptive statistics; N reflect the study sample while percentages reflect the survey-weighted data.

**Table S2.** Association between sNfL levels and type 2 DM and their combined effect on mortality after excluding participants who had cancer history at baseline in NHANES 2013–2014 (n=1896)

|  | All-cause mortality | |  | Cardiovascular mortality | |
| --- | --- | --- | --- | --- | --- |
|  | HR (95%CI) | *P* value |  | HR (95%CI) | *P* value |
| sNfL levels |  |  |  |  |  |
| Continuous sNfL | 4.42 (3.10-6.32) | <0.001 |  | 5.89 (3.70-9.38) | <0.001 |
| Not elevated | 1 [Reference] |  |  | 1 [Reference] |  |
| Elevated | 4.91 (2.46-9.79) | <0.001 |  | 12.49 (1.51-103.40) | 0.019 |
| type 2 DM |  |  |  |  |  |
| No | 1 [Reference] |  |  | 1 [Reference] |  |
| Yes | 3.18 (1.49-6.80) | 0.003 |  | 36.01 (13.57-95.57) | <0.001 |
| sNfL and type 2 DM |  |  |  |  |  |
| No type 2 DM | 1 [Reference] |  |  | 1 [Reference] |  |
| type 2 DM and not elevated | 1.47 (0.53-4.01) | 0.458 |  | 10.22 (2.26-46.09) | 0.002 |
| type 2 DM and elevated | 5.00 (2.25-11.10) | <0.001 |  | 70.42 (29.53-167.93) | <0.001 |

Abbreviations: HR, hazard ratio; CI confidence interval; sNfL, serum neurofilament light chain; DM, diabetes mellitus.

Models are adjusted for age (20-59 or ≥60), sex (male or female), and race (Non-Hispanic White, Non-Hispanic Black or Other). Elevated sNfL is characterized by values exceeding the median sNfL.

**Table S3.** Association between sNfL levels and type 2 DM and their combined effect on mortality after excluding participants who had neurologic diseases history at baseline in NHANES 2013–2014 (n=1923)

|  | All-cause mortality | |  | Cardiovascular mortality | |
| --- | --- | --- | --- | --- | --- |
|  | HR (95%CI) | *P* value |  | HR (95%CI) | *P* value |
| sNfL levels |  |  |  |  |  |
| Continuous sNfL | 4.71 (3.32-6.69) | <0.001 |  | 6.22 (4.11-9.42) | <0.001 |
| Not elevated | 1 [Reference] |  |  | 1 [Reference] |  |
| Elevated | 4.43 (2.07-9.48) | <0.001 |  | 9.62 (1.77-52.30) | 0.009 |
| type 2 DM |  |  |  |  |  |
| No | 1 [Reference] |  |  | 1 [Reference] |  |
| Yes | 3.04 (1.67-5.53) | <0.001 |  | 13.15 (2.82-61.45) | 0.001 |
| sNfL and type 2 DM |  |  |  |  |  |
| No type 2 DM | 1 [Reference] |  |  | 1 [Reference] |  |
| type 2 DM and not elevated | 1.31 (0.43-3.98) | 0.638 |  | 2.95 (0.32-26.98) | 0.337 |
| type 2 DM and elevated | 4.80 (2.70-8.52) | <0.001 |  | 25.59 (4.33-151.28) | <0.001 |

Abbreviations: HR, hazard ratio; CI confidence interval; sNfL, serum neurofilament light chain; DM, diabetes mellitus.

Models are adjusted for age (20-59 or ≥60), sex (male or female), and race (Non-Hispanic White, Non-Hispanic Black or Other). Elevated sNfL is characterized by values exceeding the median sNfL.

**Table S4.** Association between sNfL levels and type 2 DM and their combined effect on mortality after excluding participants who had CVD history at baseline in NHANES 2013–2014 (n=1888)

|  | All-cause mortality | |  | Cardiovascular mortality | |
| --- | --- | --- | --- | --- | --- |
|  | HR (95%CI) | *P* value |  | HR (95%CI) | *P* value |
| sNfL levels |  |  |  |  |  |
| Continuous sNfL | 4.23 (2.76-6.49) | <0.001 |  | 7.33 (4.90-10.96) | <0.001 |
| Not elevated | 1 [Reference] |  |  | 1 [Reference] |  |
| Elevated | 3.61 (1.56-8.39) | 0.003 |  | 25.51 (2.50-260.61) | 0.006 |
| type 2 DM |  |  |  |  |  |
| No | 1 [Reference] |  |  | 1 [Reference] |  |
| Yes | 2.82 (1.37-5.81) | 0.005 |  | 36.97 (4.75-288.02) | <0.001 |
| sNfL and type 2 DM |  |  |  |  |  |
| No type 2 DM | 1 [Reference] |  |  | 1 [Reference] |  |
| type 2 DM and not elevated | 1.21 (0.36-4.03) | 0.775 |  | 4.43 (0.33-58.77) | 0.260 |
| type 2 DM and elevated | 4.44 (2.02-9.76) | <0.001 |  | 85.68 (11.74-625.11) | <0.001 |

Abbreviations: HR, hazard ratio; CI confidence interval; sNfL, serum neurofilament light chain; DM, diabetes mellitus.

Models are adjusted for age (20-59 or ≥60), sex (male or female), and race (Non-Hispanic White, Non-Hispanic Black or Other). Elevated sNfL is characterized by values exceeding the median sNfL.
